# The global prevalence of suicidal ideation and suicide attempts among men who have sex with men: a systematic review and meta-analysis

**DOI:** 10.1186/s40001-023-01338-6

**Published:** 2023-09-21

**Authors:** Elham Nouri, Yousef Moradi, Ghobad Moradi

**Affiliations:** 1 Department of Epidemiology and Biostatistics, Faculty of Medicine, Kurdistan University of Medical Sciences, Sanandaj, Iran; 2 Social Determinants of Health Research Center, Research Institute for Health Development, Kurdistan University of Medical Sciences, Sanandaj, Iran

**Keywords:** Suicidal Ideation, Suicide Attempts, Men who have sex with men, Prevalence, High-risk behaviors

## Abstract

**Background:**

This study aimed to determine the global prevalence of suicidal ideation and attempts among men who have sex with men (MSM) as a systematic review, and meta-analysis.

**Methods:**

For this meta-analysis, a search in four international databases (PubMed, Scopus, Web of Science, and EMBASE) was designed, and performed. In the next step, the information extraction checklist was prepared based on the study authors’ opinions, and the quality of the articles was evaluated using the Newcastle–Ottawa scale (NOS) checklist. Data meta-analysis was performed using STATA16 software with a significance level below 0.05.

**Results:**

The results showed the prevalence of suicidal ideation, and suicide attempts among MSM was 21% (95% CI 17%-26%), and 12% (95% CI 8%-17%), respectively. The results of the subgroup analysis showed that the prevalence of suicidal ideation in the population of MSM living with Human immunodeficiency virus (HIV) was 40% (95% CI 35%–45%), and the prevalence of suicide attempts among MSM with HIV was 10% (95% CI 1%–27%). The prevalence of suicidal ideation in European MSM, and the prevalence of suicide attempts among American MSM were higher than other MSM in other geographical areas.

**Conclusion:**

Considering that the prevalence of suicidal ideation and attempts among these people is many times higher than that among men in the general population, developing programs for the prevention of mental disorders with special attention to suicide is necessary for these people. Screening programs are also recommended for early diagnosis and prevention of suicide among these people.

**Supplementary Information:**

The online version contains supplementary material available at 10.1186/s40001-023-01338-6.

## Introduction

Men who have sex with men (MSM) include a diverse group in terms of behaviors, identities, and health care needs [1]. According to estimates and studies, the prevalence of MSM in middle- and low-income countries in East Asia is between 3 and 5%, South and Southeast Asia between 6 and 12%, North America 3.8–6.4%, and Europe 0.03–6.5% [2, 3].

MSM are particularly vulnerable to psychiatric disorders compared to heterosexual [4, 5]. This group is often stigmatized for their sexual orientation and may experience higher rates of mental health problems, depression, and suicidal ideation or attempts [6]. Various studies have reported a prevalence of suicidal ideation among sexual minorities, particularly MSM, ranging from 10 to 55% [7–10].

Anxiety and depression are among the most important risk factors and causes of increased suicidal ideation among MSM [11]. Those who identify as sexual minorities, including MSM, and suffer from psychiatric disorders are much more likely to plan or attempt suicide than those without psychiatric disorders [8].

Suicide attempts and successful suicides are the leading causes of death among MSM [12]. Being HIV positive also increases the risk of suicidal ideation and attempts in this group [13]. Suicidal ideation occurs when an MSM becomes aware of their HIV. HIV-positive MSM may face greater stigma than their HIV-negative counterparts, which reinforces suicidal ideation [12]. Lack of finding a suitable homosexual partner, pressure from marriage with the opposite sex, as well as problems in getting used to married life are other factors that make these people think of suicide [4]. Developing a comprehensive healthcare plan that considers the unique needs of MSM is crucial in reducing mental health problems, suicidal ideation, and attempts [14].

Updating information on the prevalence of suicide (ideation, or attempts) in these groups can both provide basic information for designing interventions, and be effective in monitoring, and evaluating these interventions. This systematic review and meta-analysis aimed to determine the prevalence of suicidal ideation and attempts in the MSM in the world.

## Methods

The protocol for this study has been registered on the PROSPERO site with the code CRD42021239819. This systematic review and meta-analysis aimed to estimate the global prevalence of suicidal ideation and attempts in MSM and was conducted in the following steps:

### Search strategy and screening articles

Published articles in four databases (PubMed, Scopus, Web of Science, and EMBASE) from 1914 to 2021 were retrieved and screened.

The keywords in this review included "Suicide", and "Men who have sex with men" chosen as synonyms for the keywords from the Medical Subject Heading (MESH) search engine.

"Suicidal ideation", "Suicidal attempts", "Suicidal behaviors", "Suicide ideation", "Suicide symptoms", "Suicide attempts", "Suicidal thoughts", "Attempted Suicide", "Parasuicide", "Parasuicides", "suicide", "MSM", "Men who have sex with men", "Homosexual men", "Homosexuality", and "Homosexual".

All search syntaxs in considered international databases were mentioned in Additional file. To find gray literature, and related articles, PsycInfo, and google scholar were searched. In addition, a manual search was performed by the review of the references of related articles. After retrieving the articles, and setting the library in endnote software version 9 for each database, the articles were saved in another library in combination, and the duplicate ones were removed according to the endnote software default. Then, the remaining articles were evaluated based on their titles, abstracts, and full texts, considering the inclusion criteria. Two authors independently screened the articles based on their titles, abstracts, and full texts, and in case of any discrepancies, the results were reviewed by the study supervisor.

### Inclusion and exclusion criteria

This study aimed to determine the global prevalence of suicidal ideation and attempts among MSM. All cross-sectional studies were reviewed, and other studies (clinical trials, case ones, cohorts, letters to the editor, case reports, case studies, and review studies) were excluded. Studies in English, reporting the frequency of suicidal ideation and attempts were included in this research. Also, articles reported the suicidal ideation or attempts as an average score with standard deviation, and other indicators except the percentage or frequency were excluded from the study. So, only cross-sectional articles reported suicidal ideation or attempts as a percentage or frequency were included in the present meta-analysis. In addition, studies with a statistical population of MSM or men who have sex with men were included. However, articles with statistical populations that consisted solely of gay, bisexual, transgender, or other high-risk groups were excluded from the study.

### Data extraction

After screening, a checklist prepared with the opinion of experts was used to extract the information of these articles based on the study purpose. The checklist components included the authors’ names, study type, publication year, total sample size, country of the study, population type, age, sampling method, suicidal ideation frequency, and suicide attempt frequency.

### Risk of bias

The specified checklist that was used to assess the quality of articles was NOS [15, 16]. This checklist is appropriate for observational studies such as cross-sectional studies. Given score for each question should be done. The maximum score that may attain for articles is 9. This step was independently performed by two authors (EN and YM) and in case of any disputes, they were referred to the third researcher (GM).

### Statistical analysis

For analysis, the total sample size of the studies along with the number of MSM who had suicidal ideation or suicide attempts was extracted from all studies selected for meta-analysis. According to the extracted information, the Metaprop command was used to calculate the pooled prevalence, and the results were analyzed. Cochrane Q and I^2^ tests were used to evaluate the heterogeneity and variance between the selected studies. Funnel Plot and Egger test were used to evaluate the publication bias [17–20]. Also, meta-regression analysis and diagram were used to examine the association of the variables of the age of MSM and the sample size of the selected studies with the estimated pooled prevalence. Statistical analysis was performed using STATA 16.0, and *P*-value < 0.05 was considered.

## Results

### Qualitative results

**Fig. 1:**
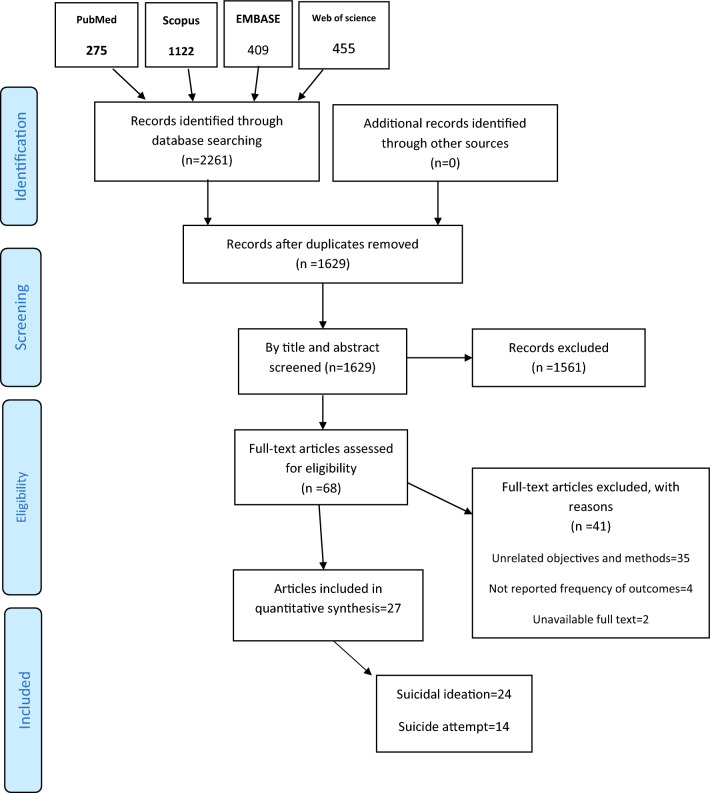
The Flowchart of Search Strategy and Syntax for suicide ideation and attempt

Initially, 2261 articles were obtained from the four databases (PubMed, Web of science, Scopus, and EMBASE), of which 275 were from PubMed, 1122 from Scopus, 409 from EMBASE, and 455 from the web of science. After removing similar items in Endnote software, 1629 articles were selected for screening their titles, and abstracts, of which 68 were reviewed for their full texts. Finally, 24 articles were included in the meta-analysis to analyze the suicidal ideation [1, 7–9, 13, 14, 21–38] and 14 ones were included to analyze suicide attempts [8, 23–25, 28, 29, 32, 33, 36–41] (Fig. 1) (Table 1). The studies were cross-sectional, and surveys while the statistical population was "men who have sex with men" or MSM. In the analysis of suicidal ideation, in 22 articles healthy MSM [1, 7–9, 13, 14, 21–28, 30–36, 38], and in 2 ones MSM with HIV were studied [29, 37]. Geographically, there were 13 studies from Asia [7, 8, 13, 14, 26, 27, 29, 31, 34–38], 7 from the Americas [1, 21–25, 28], 2 from Europe [32, 33] and 2 from Africa [9, 30]. The mean age of MSM was reported as less than 30 years in 10 studies [9, 13, 23, 25, 27, 28, 30, 34–36] and equal to more than 30 years in 11 articles [1, 7, 14, 21, 22, 26, 29, 31–33, 37] while in 3 ones, their mean age was not mentioned [8, 24, 38]. The sampling method was non-probability in 15 studies [1, 7–9, 23, 25–28, 30–32, 34, 35, 38] and unreported in 9 ones [13, 14, 21, 22, 24, 29, 33, 36, 37] (Table 2). In the analysis of suicide attempts, the population was healthy MSM in 11 studies [8, 23–25, 28, 32, 33, 36, 38, 39, 41] and HIV-positive MSM in 3 ones [29, 37, 40]. In terms of geographical areas, 6 studies were from Asia [8, 29, 36–39], 6 from the Americas [23–25, 28, 40, 41], and 2 from Europe [32, 33]. The mean age of MSM was less than 30 years in 5 studies [23, 25, 28, 36, 41], and equal to more than 30 years in 6 ones [29, 32, 33, 37, 39, 40] while their mean age was not mentioned in 3 studies [8, 24, 38]. The sampling method was non-probability in 6 studies [8, 23, 25, 28, 32, 38], and probability in 2 ones [39, 41] while it was not reported in 6 studies [24, 29, 33, 36, 37, 40] (Table 2).

**Table 1:** The characteristics of included articles of suicidal ideation and attempts

Authors –year	Year of publication	Sample size	Country	Study populations	Age [mean or median]	Method of sampling	Suicidal ideation	continents	NOS
Suicidal ideation									
Li, R.et al. 2017 [27]	2017	458	China	MSM	32	Respondent-driven sampling	23	Asia	6
Oginni, O. A.et al. 2019 [31]	2019	81	Nigeria	MSM	25	Non-random	25	Africa	7
Wang, Y. Y.et al. 2019 [53]	2019	410	China	MSM	32	NR	42	Asia	5
Hidalgo, M. A.et al. 2020 [25]	2020	448	Los Angels	MSM	NR	NR	46	America	5
Kipke, M. D.et al. 2007 [26]	2007	526	NR	MSM	20	a venue-based probability	53	America	6
Wu, Y.et al. 2015 [38]	2015	184	China	HIVMSM	31	NR	57	Asia	5
Li, R.et al. 2016 [54]	2016	547	China	MSM	30	snowball	58	Asia	5
Vu, N. T. T.et al. 2017 [36]	2017	622	Vietnam	MSM	24	convenience	61	Asia	5
Mayer, K. H.et al. 2015 [55]	2015	307	Boston	MSM	30	Respondent-driven sampling	64	America	6
Fletcher, J. B.et al. 2018 [23]	2018	286	USA	MSM	42	NR	66	America	5
Loza, O.et al. 2021 [29]	2021	150	Texas	MSM	26	snow ball	66	America	5
Sivasubramanian, M.et al. 2011 [14]	2011	150	India	MSM	25	NR	67	Asia	4
Liu, Y.et al. 2012 [28]	2012	307	China	MSM	23	convenience	73	Asia	6
Pan, X.et al. 2018 [32]	2018	454	China	MSM	33	Respondent-driven sampling	73	Asia	6
Wagner, G. J, et al. 2019 [37]	2019	226	Beirut	MSM	23	NR	75	Asia	5
Hidalgo, M. A.et al. 2015 [24]	2015	449	large Midwestern metropolitan area	MSM	19	Non-random	83	America	6
Yu, L.et al. 2018 [39]	2018	807	China	MSM	NR	Respondent-driven sampling	87	Asia	6
Sheridan, S.et al. 2009 [35]	2009	540	Vientiane	MSM	21	(VDTS)	90	Asia	5
Mo, P. K. H.et al. 2018 [30]	2018	225	China	HIVMSM	32	NR	108	Asia	5
Parker, R. D.et al. 2015 [33]	2015	265	Estonia	MSM	31	Non-random	118	Europe	6
Rüütel, K.et al. 2017 [34]	2017	265	Estonia	MSM	32	NR	119	Europe	5
Mu, H.et al. 2016 [8]	2016	807	China	MSM	NR	Respondent-driven sampling	148	Asia	7
Stahlman, S.et al. 2016 [9]	2016	1555	African country	MSM	23	Respondent-driven sampling	202	Africa	6
Biello, K. B.et al. 2016 [22]	2016	1752	Latin America	MSM	35	NR	223	America	5
Suicidal attempts									
Mo, P. K. H.et al. 2018 [30]	2018	225	China	HIVMSM	32	NR	6	Asia	5
Wu, Y.et al. 2015 [38]	2015	184	China	HIVMSM	31	NR	10	Asia	5
Hidalgo, M. A.et al. 2020 [25]	2020	448	Los Angeles	MSM	NR	NR	17	America	5
Yu, L.et al. 2018 [39]	2018	807	China	MSM	NR	Respondent-driven sampling	24	Asia	6
Loza, O.et al. 2021 [29]	2021	150	Texas	MSM	26	snowball	26	America	5
Rüütel, K.et al. 2017 [34]	2017	265	Estonia	MSM	32	NR	29	Europe	5
Parker, R. D.et al. 2015 [33]	2015	265	Estonia	MSM	31	Non-random	29	Europe	6
Wagner, G. J, et al. 2019 [37]	2019	226	Beirut	MSM	23	NR	33	Asia	5
Mu, H.et al. 2016 [8]	2016	807	China	MSM	NR	Respondent-driven sampling	37	Asia	7
Kipke, M. D.et al. 2007 [26]	2007	526	-	MSM	20	a venue-based probability	44	America	6
Pantalone, D. W.et al. 2018][41]	2018	166	USA	HIVMSM	44	NR	46	America	6
Remafedi, G.et al. 2002 [42]	2002	254	Minnesota	MSM	20	random	85	America	7
Hidalgo, M. A.et al. 2015 [24]	2015	449	large Midwestern metropolitan area	MSM	19	Non-random	174	America	6
Hidaka, Y.et al. 2014 [40]	2014	5731	Japan	MSM	30	Non-representative	805	Asia	6

**Table 2:** The pooled prevalence of suicidal ideation and attempts among MSM (overall prevalence, subgroup analysis of suicidal ideation and attempts)

Suicidal ideation		No. study(SS)	No. suicide ideation	Pooled prevalence	Heterogeneity assessment		
					I2 %	*p*-value	Test Q
Over all		24 (11,821)	2027	21% (17–26)%	96.88	0.00	737.85
population	Healthy	22(11,412)	1862	20% (16–24)%	96.50	0.00	600.54
	HIV	2(409)	165	40% (35–45)%	–	–	–
continent	Asia	13 (5737)	962	20% (14–26)%	96.83	0.00	378.18
	America	7 (3918)	601	19% (13–25)%	94.84	0.00	116.34
	Europe	2 (530)	237	45% (40–49)%	–	–	–
	Africa	2 (1636)	227	14% (12–15)%	–	–	–
age	> 30	10 (4606)	795	23% (17–30)%	96.10	0.00	230.64
	= < 30	11 (5153)	951	23% (15–31)%	97.86	0.00	466.39
	NR	3 (2062)	281	13% (8–19)%	–	–	–
NOS score	0–3	0(0)	–	–	–	–	–
	4–6	22(10,933)	1854	21% (17–26)%	92.03	0.00	724.92
	7–9	2(888)	173	19%(17–22)%	–	–	–

Suicidal attempts		No. study(SS)	No. suicide ideation	Pooled prevalence	Heterogeneity assessment		
					I2 %	*p*–value	Test Q
Over all		14 (10,503)	1365	% 12 (8–17)%	97.68	0.00	561.02
population	Healthy	11(9928)	1303	% 13 (8–19)%	97.97	0.00	493.31
	HIV	3 (575)	62	%10 (1–27)%	–	–	–
continent	Asia	6 (7980)	915	% 7 (3–12)%	97.70	0.00	217.28
	America	6 (1993)	392	% 20 (8–35)%	98.25	0.00	286.28
	Europe	2 (530)	58	% 11 (8–14)%	–	–	–
age	> 30	5 (1605)	362	% 21 (10–36)%	97.59	0.00	165.91
	= < 30	6 (6836)	925	% 11 (6–16)%	93.55	0.00	77.55
	NR	3 (2062)	78	% 4 (3–5)%	–	–	–
NOS score	0–3	0 (0)	–	–	–	–	–
	4–6	12 (9442)	1243	% 12 (7–17)%	97.43	0.00	428.54
	7–9	2 (1061)	122	9% (8–11)%	–	–	–

## Quantitative results

### Suicidal ideation

#### Prevalence of suicidal ideation among MSM

**Fig. 2:**
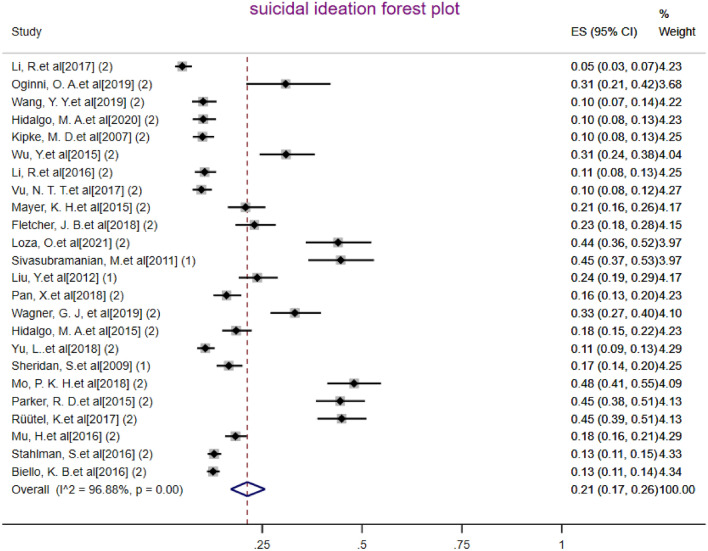
The pooled prevalence of Suicidal ideation in MSM

The sample size of MSM in the total of 24 articles was 11,821 people whereas the frequency of whom with suicidal ideation was 2027 people (Table 2). After combining these studies, the pooled prevalence of suicidal ideation in MSM in the world was 21% (95% CI 17%–26%) with heterogeneity (I^2^) of 96.88% (Fig. 2). The prevalence range in the studied articles varied from 5 to 48% while the lowest prevalence equal to 5% (95% CI 3–7%) was related to the study of Li et al. [26] and the highest prevalence equal to 48% (95% CI 41–55%) was related to the study of Mo et al.[29].The Eggers test results showed publication bias occurred in calculating the pooled prevalence of suicidal ideation in MSM (*B* = 9.85, SE = 0.455, *P* < 0.001). To show the publication bias, the funnel plot was used (Fig. 4). In meta-regression analysis, the effect of age on prevalence of suicidal ideation was analyzed, the results of which are shown in Fig. 4. According to the results of this analysis, age was not a significant effect with the prevalence of suicidal ideation in MSM (coefficient = 0.0018, SE = 0.0055, *P* = 0.738). The publication year of the studies also had no significant effect with the prevalence calculated in the meta-analysis (coefficient = 0.0036, SE = 0.0085, *P* = 0.677).

### Subgroup analysis of suicidal ideation in MSM

#### Subgroup analysis based on the population type

The results of subgroup analysis of the population type showed that MSM participants in 22 studies were healthy while those in 2 studies were infected by HIV. The sample size of healthy MSM was 11,412 people of whom 1862 had suicidal thoughts. The pooled prevalence of suicidal ideation among them was 20% (95% CI 16%-24%) with heterogeneity (I2) of 96.50%. Also, in two studies, the sample size of MSM with HIV was 409 people of whom 165 had suicidal ideation. The pooled prevalence of suicidal ideation among them was 40% (95% CI 35%–45%) (Table 2).

### Subgroup analysis based on the continent

The results of subgroup analysis of different geographical areas showed that the prevalence of suicidal ideation among MSM varied from 14 to 45%. The sample size of Asian MSM in 13 studies was 5737 people of whom 962 had suicidal ideation with a pooled prevalence of 20% (95% CI 14%–26%) and heterogeneity (I2) of 96.83%. Also, in 7 studies, the sample size of American MSM was 3918 people of whom 601 had suicidal ideation. The pooled prevalence of suicidal ideation among American MSM was 19% (95% CI 13%–25%) with heterogeneity (I2) of 94.84% (Table 2).

### Subgroup analysis based on age

The results showed that in 10 studies, the mean age of MSM was less than 30 years. Out of the sample size of 4606 people, 795 had suicidal ideation with a pooled prevalence of 23% (95% CI 17%–30%). The sample size in 11 studies with the mean age of 30 years and more was 5153 MSM of whom 951 had suicidal ideation.

### Subgroup analysis based on the NOS score

22 studies with a sample size of 10,933 people had the NOS scores in the range of 4–6. The pooled prevalence of suicidal ideation in these studies was 21% (95% CI 17%–26%). Two studies with a sample size of 888 MSM had an NOS score of 7. The pooled prevalence of suicidal ideation in these studies was 19% (95% CI 17%—22%) (Table 2).

### Suicide attempts

#### Prevalence of suicide attempts among MSM

**Fig. 3:**
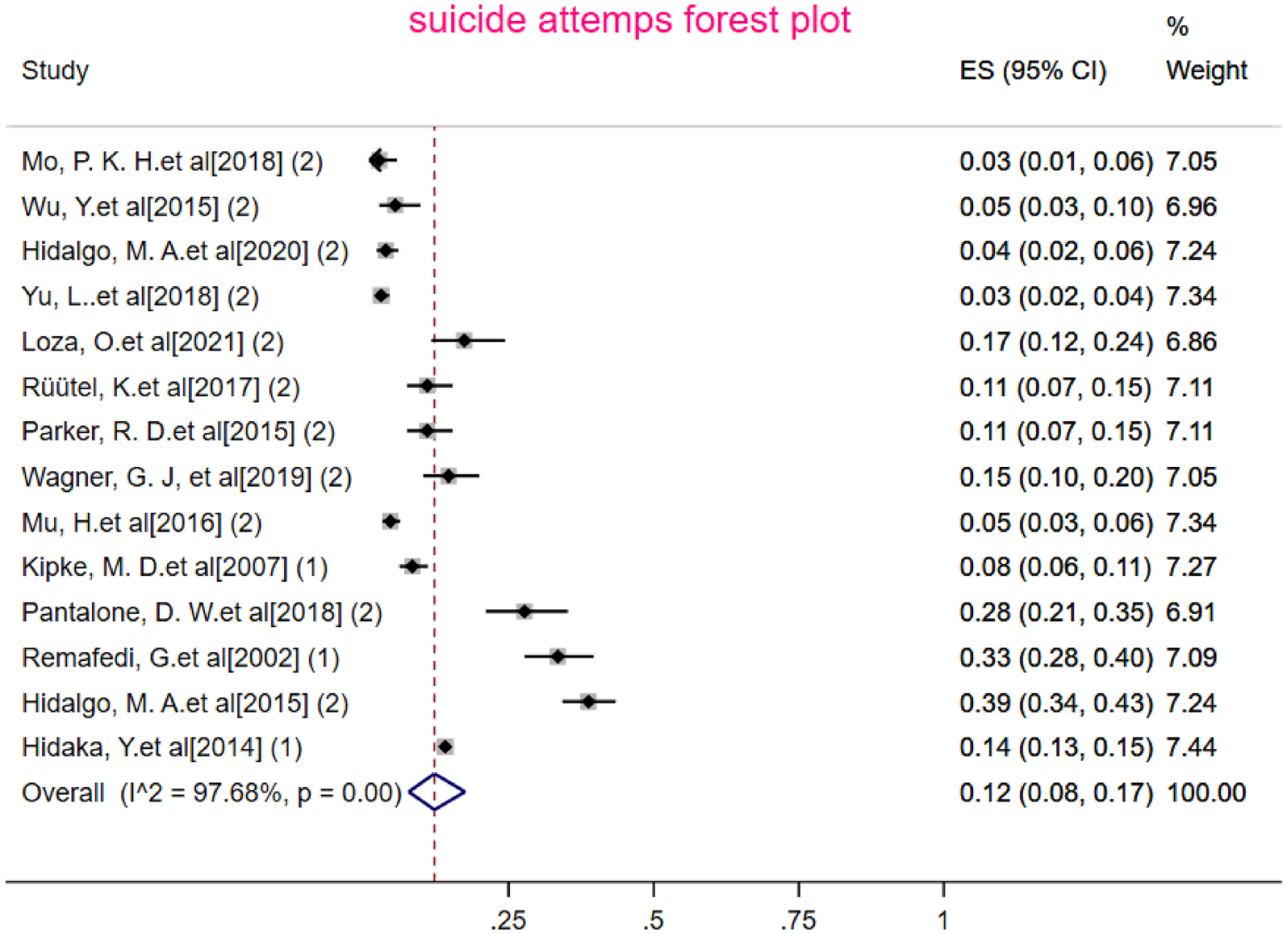
The pooled prevalence of Suicide attempts in MSM

**Fig. 4:**
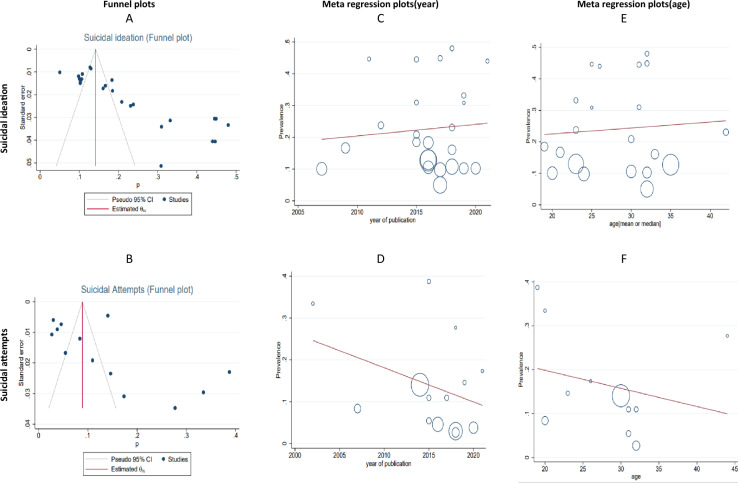
**A** and **B**: results of publication bias in pooled prevalence of suicidal ideation and attempt, **C** and **D**: meta regression of pooled prevalence and year (suicidal ideation and attempts), **E** and **F**: meta regression of pooled prevalence and age (suicidal ideation and attempts)

The sample size of MSM in a total of 14 articles (Table 2) was 10,503 people, of whom 1365 had attempted suicide. After combining these studies, the pooled prevalence of suicide attempts in MSM in the world was 12% (95% CI 8–17%) with heterogeneity (I2) of 97.68% (Fig. 3). The prevalence range in the studied articles varied from 3 to 39% with the lowest prevalence of 3% related to the study of Mo et al. [29] (95% CI 1–6%), and the study of Yu et al. [38] (95% CI 2–4%). Also, the highest prevalence of 39% was related to the study of Hidalgo M.A. et al. (95% CI 34–43% ) [23] (Table 2). The results of the Eggers test showed that the publication bias in calculating the pooled prevalence of suicide attempts in MSM occurred (B = 3.63, SE = 0.481, *P* < 0.001). To show the publication bias, the funnel plot was used, which is shown in Fig. 4. Also, to investigate the association between the age of MSM and the year of publication of the studies included in the meta-analysis, meta-regression analysis was used, which is shown in Fig. 4. According to the results of this analysis, age was not significantly affected with the pooled prevalence of suicide attempts in MSM (coefficient =  0.004134, SE = 0.00511, *P* = 0.44). The publication year of the studies also had no significant effects with the pooled prevalence calculated in the meta-analysis (coefficient =  0.00815, SE = 0.0061, *P* = 0.209).

### Subgroup analysis of suicide attempts

#### Subgroup analysis based on the population type

The results of subgroup analysis based on the population type showed in 11 studies healthy MSM had participated while in 3 ones, participants were infected by HIV. The sample size of healthy MSM was 9928 people of whom 1303 had suicide attempts. The pooled prevalence among them was 13% (95% CI 8% –19%) with heterogeneity (I2) of 97.97%. Also, in 3 studies, the sample size of MSM with HIV was 575 people of whom 62 had attempted suicide. The pooled prevalence of suicide attempts among them was 10% (95% CI 1%–27%) (Table 2).

### Subgroup analysis based on the continent

The results of subgroup analysis of different geographical areas showed the prevalence of suicide attempts among MSM ranged from 7 to 20%. The sample size of Asian MSM in 6 studies was 7980 people of whom 915 had attempted suicide with a pooled prevalence of 7% (95% CI 3%–12%) and heterogeneity (I2) of 97.70%. Also, in 6 studies, the sample size of American MSM was 1993 people of whom 392 had suicide attempts. The pooled prevalence of suicide attempts among American MSM was 20% (95% CI 8%–35%) with heterogeneity (I2) of 98.25%. Two studies had a sample size of 530 European MSM of whom 58 had suicide attempts. The pooled prevalence of suicide attempts among European MSM was 11% (95% CI 8%–14%) (Table 2).

### Subgroup analysis based on age

The results showed in 5 studies, the mean age of MSM was less than 30 years. Out of the sample size of 1605 people, 362 had attempted suicide with a pooled prevalence of 21% (95% CI 10%–36%). The sample size in 6 studies with a mean age of 30 years and more was 6836 MSM of whom 925 had attempted suicide. The pooled prevalence of suicide attempts at this mean age was 11% (95% CI 6%–16%) (Table 2).

### Subgroup analysis based on the NOS score

Twelve studies with a sample size of 9442 MSM had the NOS scores in the range of 4–6. The pooled prevalence of suicide attempts in these studies was 12% (95% CI 7%–17%). Two studies with a sample size of 1061 MSM had a NOS score of 7. The pooled prevalence of suicide attempts in these studies was 9% (95% CI 8%–11%) (Table 2).

## Discussion

The results showed the pooled prevalence of suicidal ideation and attempts among MSM were 21% and 12%, respectively. These results showed the prevalence of suicidal ideation and attempts were higher in MSM as a group of sexual minorities compared to the general population.

The prevalence of suicidal ideation and suicide attempts in the general population have been investigated by several studies, including those conducted by Cao, Xiao-Lan (2015), Bifftu and Berhanu Boru (2021), and Castillejos and Ma Carmen (2021). Their results indicated that the prevalence of suicidal ideation was 3.9%, 9%, and 9.8%, respectively, while the prevalence of suicide attempts was 0.8%, 4%, and 2.8%, respectively [42–44]. MSM are at a higher risk of experiencing suicidal ideation and attempt than the general population, which is not surprising given the unique challenges and stressors they may face [45].

MSM individuals may experience suicidal ideation and suicide attempts as a result of factors such as their sexual orientation, challenges in finding same-sex partners, discrimination based on gender, and lower levels of social acceptance for mental health issues [4]. The high rate of suicide in a study that conducted by Kohlbrenner has been attributed to perceived discrimination due to sexual orientation, and its extent [46]. One study found MSM suffering from any psychiatric disorder were 4 to 7 times more likely to think or attempt suicide than MSM with no psychiatric disorder [8].

The results of the present meta-analysis showed suicidal ideation was equally prevalent in the age group of less than 30 years, and 30 years and older, which was estimated at 23% while suicide attempts were higher in the age group of less than 30 years with a prevalence of 21% than the age group of 30 years and more with a prevalence of 11%. MSM of all ages are at a heightened risk for experiencing suicidal ideation and suicide attempts, largely due to the stigma and psychological distress associated with their sexual minority status. Additionally, a positive correlation has been observed between a history of childhood sexual abuse or distress and suicide attempts among individuals who identify as sexual minorities [47].

Previous research has demonstrated that experiences of victimization related to race and sexuality were strongly associated with higher levels of psychological distress and increased risk of suicide attempts among young MSM who identified as African–American, Latino, or multiracial [48]. On the other hand, with the disclosure of their sexual identity among their peers, and families as well as because of social exclusion and stigma, these people experience more mental disorders from an early age than their other peers, which gives rise to suicidal ideation in them. Younger age groups are more likely to commit suicide because they are more prone to sexual abuse and are less tolerant of stigma than older age groups. Young MSM also suffers from depression due to lack of social support and isolation [49–51].

According to the findings of the meta-analysis, Asian and American MSM have a similar prevalence of suicidal ideation. Furthermore, the study by Mathy RM found that there is a significant association between sexual orientation and suicidal ideation in Asia, North America, and South America. However, in Europe, there was no significant association between sexual orientation and suicidal ideation. The results also suggest that MSM in the Americas reported a higher history of suicidal ideation than heterosexuals. These findings could be useful in developing targeted interventions and prevention strategies to address the mental health needs of MSM, particularly in regions where a significant association between sexual orientation and suicidal ideation has been identified [52].

In the present study, Europe had the highest prevalence of suicidal ideation with a rate of 45%. However, conflicting results have been reported in Robin M. Mathy's study. The study found there was less gender discrimination in Europe due to freer sexual attitudes as well as culture-building in the continent's media [52]. The reason for the increase in suicidal ideation in European MSM in the present meta-analysis was due to the results of other studies from Europe, which showed the high prevalence of suicidal ideation in this region.

In addition, results of this meta-analysis showed the pooled prevalence of suicidal ideation, and attempts among MSM with HIV was 40%, and 10%, respectively. The results of a study conducted by Gizachew KD on the population of hospitalized people living with HIV/AIDS showed the prevalence of suicidal ideation, and suicide attempts were 16% and 7.1%, respectively, which contradicted the present study results [53]. Individuals who fear that their sexual partners may refuse to use condoms may engage in high-risk unprotected sexual behaviors, which can increase the transmission of HIV and lead to heightened psychological distress, including suicidal ideation [54]. In addition, MSM individuals may experience stigma related to HIV, which can further exacerbate their suicidal ideation.

This meta-analysis is the first one to examine the prevalence of suicidal ideation and attempts with this number of articles on the MSM in the world. The study also aimed to evaluate the prevalence of suicidal ideation and attempts in both healthy MSM and MSM infected with HIV separately, which could provide a more comprehensive understanding of the issue. By examining the prevalence of suicidal ideation and attempts in MSM, the proposed meta-analysis could shed light on the mental health challenges faced by this vulnerable population. It may also highlight the importance of providing appropriate support and intervention strategies that are tailored to the specific needs of MSM, as well as the need for further research in this area. Additionally, the findings of this meta-analysis could potentially inform public health policies and interventions aimed at reducing suicide rates among MSM.

One of the limitations of this study was the lack of access to studies to determine the rate of successful suicides among MSM. Thus, we could not provide an estimate. We can also point to the small number of studies in Europe, and Africa for more accurate inference from the results. Some studies did not mention the mean age of the participants. Furthermore, because this meta-analysis focused on determining prevalence, only cross-sectional studies were included in the analysis. This led to high heterogeneity in the results when the studies were combined, which is a common limitation of all meta-analyses that aim to determine prevalence.

## Conclusion

The results of the present study showed suicidal ideation, and suicide attempts were several times higher among MSM than men in the general population. By screening MSM and early diagnosis of mental disorders, suicidal ideation well as suicide attempts can be reduced among them. For this reason, it is necessary to develop special programs for these people to prevent mental disorders with special attention to suicide prevention.

### Supplementary Information

**Additional file 1. **Query syntax in all international databases.

## Data Availability

The data extracted for analyses are available by the corresponding author upon reasonable requests.

## Abbreviations

MSM: Men who have Sex with Men
NOS: Newcastle–Ottawa Scale
HIV: Human immunodeficiency virus
MESH: Medical Subject Headings
