# Supplementary material for: The global prevalence of suicidal ideation and suicide attempts among men who have sex with men: a systematic review and meta-analysis
Source: Eur J Med Res. 2023 Sep 21;28:361. doi: 10.1186/s40001-023-01338-6 (PMC10514985; doi:10.1186/s40001-023-01338-6)
Supplement: Supplementary file 1 — Additional file 1. Query syntax in all international databases. [file 40001_2023_1338_MOESM1_ESM.docx]

**The global prevalence of suicidal ideation and suicide attempts among men who have sex with men: A systematic review and meta-analysis**

Supplementary File

[Search Strategy 2](#_Toc102687603)

# Search Strategy

| Pubmed search strategy |
| --- |
| ("Suicidal ideation"[Title/Abstract] OR "Suicidal attempt"[Title/Abstract] OR "Suicidal behaviors"[Title/Abstract] OR "Suicide ideation"[Title/Abstract] OR "Suicide symptoms"[Title/Abstract] OR "Suicide attempts"[Title/Abstract] OR "Suicidal thoughts"[Title/Abstract] OR "Attempted Suicide"[Title/Abstract] OR "Parasuicide"[Title/Abstract] OR "Parasuicides"[Title/Abstract] OR "suicide"[Title/Abstract]) AND ("MSM"[Title/Abstract] OR "Men who have sex with men"[Title/Abstract] OR "Homosexual men"[Title/Abstract] OR "Homosexuality"[Title/Abstract] OR "homosexual"[Title/Abstract]) |

| Scopus search strategy |
| --- |
| ( TITLE-ABS-KEY ( "Suicidal ideation"  OR  "Suicidal attempt"  OR  "Suicidal behaviors"  OR  "Suicide ideation"  OR  "Suicide symptoms"  OR  "Suicide attempts"  OR  "Suicidal thoughts"  OR  "Attempted Suicide"  OR  "Parasuicide"  OR  "Parasuicides"  OR  "suicide" )  AND  TITLE-ABS-KEY ( msm  OR  "Men who have sex with men"  OR  "Homosexual men"  OR  "Homosexuality"  OR  "homosexual" ) ) |

| EMBASE search strategy |
| --- |
| ('suicidal ideation'/exp OR 'suicidal ideation' OR 'suicidal attempt'/exp OR 'suicidal attempt' OR 'suicidal behaviors' OR 'suicide ideation'/exp OR 'suicide ideation' OR 'suicide symptoms' OR 'suicide attempts' OR 'suicidal thoughts'/exp OR 'suicidal thoughts' OR 'attempted suicide'/exp OR 'attempted suicide' OR 'parasuicide'/exp OR 'parasuicide' OR 'parasuicides' OR 'suicide'/exp OR 'suicide') AND (msm:ab,ti OR 'men who have sex with men':ab,ti OR 'homosexual men':ab,ti OR 'homosexuality':ab,ti OR 'homosexual':ab,ti) |

| Web of Science search strategy |
| --- |
| TOPIC: ("Suicidal ideation" OR "Suicidal attempt" OR "Suicidal behaviors" OR "Suicide ideation" OR "Suicide symptoms" OR "Suicide attempts" OR "Suicidal thoughts" OR "Attempted Suicide" OR "Parasuicide" OR "Parasuicides" OR "suicide") AND TOPIC: (MSM OR "Men who have sex with men" OR "Homosexual men" OR "Homosexuality" OR "homosexual") |
